# A Laundry Maid and a Hospital Epidemic

**Published:** 1923-07

**Authors:** 


### A LAUNDRYMAID AND A HOSPITAL EPIDEMIC.

HOSPITALS should be, and usually are, centres for learning and healing, and thanks to the work of Holmes, Semmelweis, Pasteur and Lister, maternity hospitals are no longer regarded as hot-beds of infection. But now and then epidemics still break out in these hospitals, and Dr. H. Schultheiss, of Basle, has recently reported an epidemic of pemphigus with a moral attaching to it. Late in March last year two babies in different wards developed a rash characteristic of pemphigus. Though these cases occurred simultaneously, a serious epidemic was not feared, and no special precautions were taken to arrest further spread of the disease. Soon other cases occurred, and there were altogether 21. Numerous precautions were taken, the infected infants were isolated, special nurses were detailed for their exclusive nursing, the use of a common bathroom for the infants was discontinued, the bath water was medicated with antiseptics, and various other measures were taken but without the slightest result. The epidemic continued to spread to different wards and different storeys in the same building, and cases occurred in both the public and the private wards. The pemphigoid vesicles were invariably found to contain one and the same germ—the *staphylococcus aureus*. A search was made for carriers, but none of the hospital staff had an incriminating sore throat or septic wound.

"GREAT EFFECTS FROM LITTLE CAUSES SPRING."

When the epidemic had lasted nearly a fortnight, and the search for a septic focus had left the authorities baffled, an important clue was found. A bandage, which had been secured round the breasts of a woman for some days, was removed, revealing two characteristic impetiginous eruptions of the size of a small coin. The skin had been perfectly clean before the bandage had been secured, and the suspicion arose that the bandage had been infected at the laundry. The staff of the laundry had to submit to a searching examination, and a laundry maid was found to have a bad whitlow of the right index finger. For at least a week she had been sorting linen without any dressing on this finger, and for an even longer period it had evidently been septic. The peccant maid was removed, and all the suspect linen was withdrawn from use and sterilised. No new cases occurred, and the epidemic died out as rapidly as it had started. Hospital epidemics like this are by no means rare, and the moral of this tale is that a search for the offending focus of infection should not be limited to doctors, nurses, ward maids and the like, but should be extended to the periphery of the hospital world, and should not be abandoned till the infection has been traced to its source.

### River Trips for the Wounded.

River trips for the wounded men still in the London hospitals are being continued this year. A sum of £1,000 is needed to carry through the programme, and gifts should be sent to the Rev. John Stanley, Westminster House, Durand Gardens, Stockwell, S.W.9.

